# Does cerclage improve neonatal outcomes in a molar pregnancy and a coexistent fetus? a case report

**DOI:** 10.1186/1756-0500-5-621

**Published:** 2012-11-05

**Authors:** Eduardo Aguin, Victor Aguin, Ligia Cisneros, Tina Aguin, Cosmas Van de ven, Ray Bahado-Singh

**Affiliations:** 1 Department of Obstetrics and Gynecology, Division of Maternal Fetal Medicine, University of Michigan, Ann Arbor, MI 48109, USA; 2 Department of Obstetrics and Gynecology, Wayne State University, Detroit, MI 48201, USA

**Keywords:** Cerclage, Molar pregnancy, Neonatal outcome

## Abstract

**Background:**

Complete hydatiform mole and coexistent viable fetus is very rare. The use of a cervical cerclage for cervical indications in the presence of this condition has never been reported. Although the diagnosis was made postnatal, the objective is to present a case with good neonatal outcome.

**Case presentation:**

A patient presented with vaginal spotting around 23 weeks. She has a history of four preterm deliveries. Her cervix was dilated and a cerclage was placed. She presented again with PPROM around 25 weeks. She went into spontaneous preterm labor and delivered a viable fetus that is a healthy girl today. Eventually the pathology of the placenta showed a complete hydatidiform mole.

**Conclusion:**

It is necessary to inform patients about the potential risks and poor outcomes of this condition. For those who desire all potential interventions, cerclage placement could be considered.

## Background

A twin pregnancy with a complete hydatidiform mole and a coexistent fetus (CHCF) occurs in approximately 1 in 22,000 to 100,000 pregnancies [1,2]. The probability of carrying a CHCF to term is low due to the risks of spontaneous abortion, preterm delivery, hyperthyroidism, preeclampsia, vaginal bleeding, thrombolytic disease, and persistent gestational trophoblastic neoplasia [3]. Early termination of pregnancy has often been advised in the past. The course of management of this unusual twin pregnancy still remains unclear.

Prematurity is clearly a potential risk associated with CHCF. Despite previously published data that demonstrated the inefficacy of cervical cerclage, it still remains as one of the potential alternatives that can be used to decrease prematurity associated with cervical variables [4].

The use of cervical cerclage in a patient with presence of complete hydatidiform mole and a coexistent viable fetus has never been reported.

## Case presentation

The patient was a 27-year-old gravida 7 para 4 who presented with vaginal spotting at 23 weeks and 2 days of gestation. Her obstetric history included 4 preterm deliveries and 1 spontaneous abortion at 16 weeks. Her cervix was 4–5 cm dilated with a bulging bag. Ultrasound showed a live pregnancy with breech presentation, as well as a placental mass with differential diagnosis of a chorioangioma measuring 10x40 mm. Cervical length was unable to be accurately measured secondary to the anatomical changes. The patient was counseled and she consented to undergo amniocentesis and rescue cerclage placement. The amniocentesis showed a karyotype of 46XX. A cerclage was placed using the McDonald technique. The patient also received betamethasone for fetal lung maturity secondary to the risks for preterm delivery. The patient was discharged in stable condition after 2 days. Tocolytics or progesterone was not used.

She returned with vaginal bleeding and premature rupture of the membranes at 25 weeks and 2 days. The potential benefit of retaining the cerclage in order to prolong latency and decrease complications related to prematurity was considered. The decision was made to remove the cerclage at 25 weeks and 6 days due to the presence of heavy vaginal bleeding and the suspicion of preterm labor. She went into spontaneous preterm labor and vaginal delivery of a viable female fetus weighting 625 grams. The delivery was uneventful; however, the placenta was partially retained after delivery. An exploration of the uterine cavity and cervix was performed. This was followed by curettage due to continued heavy bleeding. Grape-like structures were seen during the procedure.

The placenta weighted 200 grams. The tissue appeared to have two distinct areas, one with normal placental tissue and one mixed with vesicles. This vesicular placental tissue was reported by pathology to represent gestational trophoblastic disease. Today, the fetus discussed previously is a healthy 4-year-old girl without major medical problems, aside from mild intermittent asthma. Her mother had a beta-hCG level less than 5 mIU/ml at 8 weeks post partum and is full remission today.

## Conclusions

A dilemma in the management of CHCF is whether to follow them expectantly or to terminate the pregnancy. With a partial mole and coexisting live fetus, there is a high chance of fetal malformation and growth restriction because of associated triploidy [5]. Also, patients need to be counseled about all possible risks associated with CHCF, including the potential evolution to a gestational trophoblastic neoplasia [6]. The incidence of CHCF is increasing with in vitro fertilization, ovulation stimulation, and other fertility procedures [7]. In this unique case, the diagnosis of CHCF was made following delivery. Although cytogenetic studied were not performed, the karyotype was 46XX with a histology report supporting CHCF. However, in most situations, CHCF will be diagnosed after delivery as diagnosis can be reached only by invasive methods [8]. We should consider the possibility of CHCF in patients with vaginal bleeding and an ultrasound abnormality suggestive of a placental mass. If there is a strong suspicion of CHCF, an amniocentesis should be performed to rule out triploidy associated with a partial hydatidiform mole.

In conclusion, a detailed explanation and discussion about the risks and poor outcomes should be provided to the patient. Informed consent for interventions that may prolong the pregnancy should be obtained. A patient who desires to continue with the pregnancy needs close surveillance for early detection of maternal complications. The patient should be followed with ultrasonography and beta-hCG level measurements [7]. Pertinent tests to rule out hyperthyroidism, pre-eclampsia, thrombolytic disease, as well as cervical length should be considered. Furthermore, regardless of the obstetrical risk associated with CHCF, our recommendation is to be prepared for possible procedures, such as a rescue cerclage for cervical indications. Our patient had a questionable history of cervical insufficiency, and advanced cervical dilation. Rescue cerclage was performed and the pregnancy continued until viability was reached. This unique pregnancy resulted in a healthy mother and child.

## Consent

Written informed consent was obtained from the patient for the publication of this case report.

## Competing interest

None of the authors have a conflict of interest. No financial support, supplies or services were received from a commercial organization. No funding was received from the National Institutes of Health or any other institute.

## Authors’ contributions

All authors read and approved the final manuscript.
